# Cellular immune response to hepatitis-C-virus in subjects without viremia or seroconversion: is it important?

**DOI:** 10.1186/s13027-016-0070-0

**Published:** 2016-05-16

**Authors:** Sayed F. Abdelwahab

**Affiliations:** Departement of Microbiology and Immunology, Faculty of Medicine, Minia University, Minia, 61511 Egypt; Department of Microbiology, College of Pharmacy, Taif University, Taif, 21974 Kingdom of Saudi Arabia

**Keywords:** Cell-mediated immunity, CD4 T cells, CD8 T cells, Hepatitis C virus, Healthcare workers, Interferon-γ, IVDU, T-cells, Viral hepatitis, Vaccines

## Abstract

Hepatitis C Virus (HCV) causes chronic infection and represents a global health burden. To date, there is no licensed vaccine for HCV. The high viral replication rate and the existence of several HCV genotypes and quasispecies hamper the development of an effective universal vaccine. In this regard, the current HCV vaccine candidates show genotype-specific protection or narrow cross reactivity against other genotypes. Importantly, HCV spontaneous clearance occurs in 15–50 % of infected subjects, indicating that natural resistance to chronic infection exists. This phenomenon was demonstrated among humans and chimpanzees and continues to motivate researchers attempting to develop an effective HCV vaccine. However, what constitutes a protective immune response or correlate of protection against HCV infection is still vague. Additionally, the mechanisms behind successful HCV clearance suggest the coordination of several arms of the immune system, with cell-mediated immunity (CMI) playing a crucial role in this process. By contrast, although neutralizing antibodies have been identified, they are isolate-specific and poorly correlate with viral clearance. Antigen-specific CD4 T cells, instead, correlate with transient decline in HCV viremia and long-lasting control of the infection. Unfortunately, HCV has been very successful in evading host immune mechanisms, leading to complications such as liver fibrosis, cirrhosis and hepatocellular carcinoma. Interestingly, CMI to HCV antigens were shown among exposed individuals without viremia or seroconversion, suggesting the clearance of prior HCV infection(s). These individuals include family members living with HCV-infected subjects, healthcare workers, IV drug users, and sexual contacts. The correlates of protection could be closely monitored among these individuals. This review provides a summary of HCV-specific immune responses in general and of CMI in particular in these cohorts. The importance of these CMI responses are discussed.

## Background

**Fig. 1:**
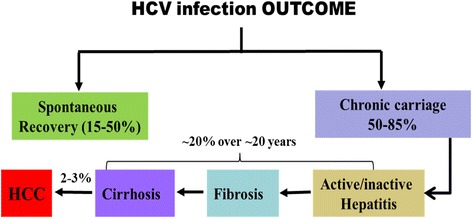
Outcome of HCV infection. Different outcomes of HCV infection and the different possibilities are shown

**Fig. 2:**
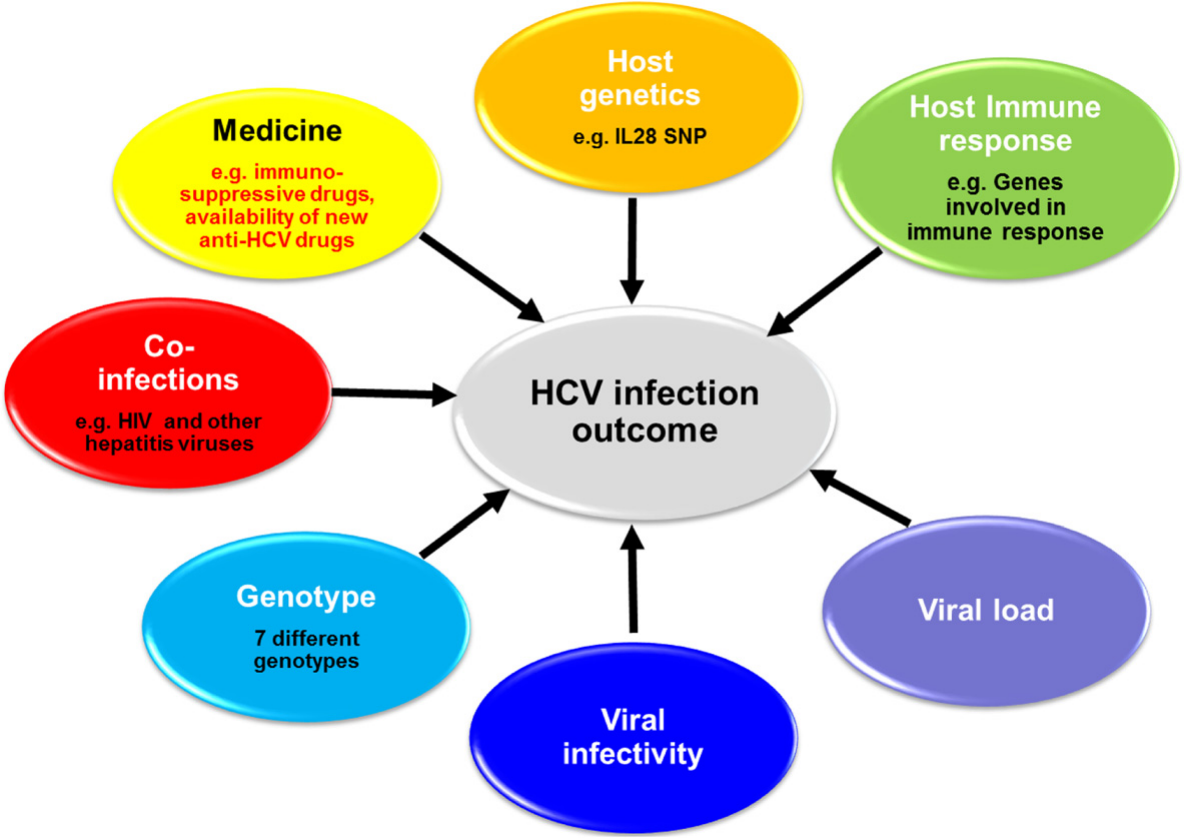
Factors affecting HCV natural history. Host factors are shown in the upper part of the figure and viral factors are shown in the lower part of the figure. Coinfections and comorbidities also contribute to the outcome of infection

Hepatitis C virus (HCV) infection is a global health burden. Nearly 185 million subjects (~3 %) of the world’s population are affected by this virus. Liver cirrhosis, progressing liver disease, and hepatocellular carcinoma (HCC) are common complications of chronic HCV infection [1, 2]. Primary infections with HCV are usually asymptomatic [3], and the majority of cases develop chronic infection. Approximately 15–50 % of infected individuals undergo spontaneous viral clearance [4]. Figure 1 shows the possible outcomes of HCV infection. Viral and host factors such as gender, coinfections, and genetics are known to affect the likelihood of clearance or persistence [5]. Figure 2 shows a summary of the factors contributing to the different outcomes of HCV infection. For example, interleukin 28B (IL28B, also known as interferon lambda 3) single nucleotide polymorphisms (SNP) have been used as predictors of viral clearance with and without therapy [6–8]. In this regard, infected patients with the IL28B.rs12979860 CC “favorable” allele are more likely to spontaneously clear HCV infection and respond more favorably to interferon (IFN)-α treatment [6–8]. Natural protective immunity against HCV has also been proven in both humans [9, 10] and chimpanzees [11]. Importantly, host-specific cell-mediated immunity (CMI) plays an essential role in the control of HCV infection [12–14].

New vaccine strategies may benefit from targeting the generation of potent high-avidity CD8 T-cell responses which can clear virus-infected cells at mucosal surfaces. Such T-cell responses could potentially prevent mucosal transmission and significantly restrict the development of chronic HCV infection. Importantly, HCV-specific memory CD4 and CD8 T cells were shown to persist for approximately 20 years after viral clearance among humans [15] and also up to 7 years among chimpanzees [13]. The mechanisms behind this phenomenon and how memory responses are maintained are not well understood. Knowledge of factors that affect the differentiation of long-lived effector and central memory cells are important for the development of an effective T-cell vaccine [16].

**Fig. 3:**
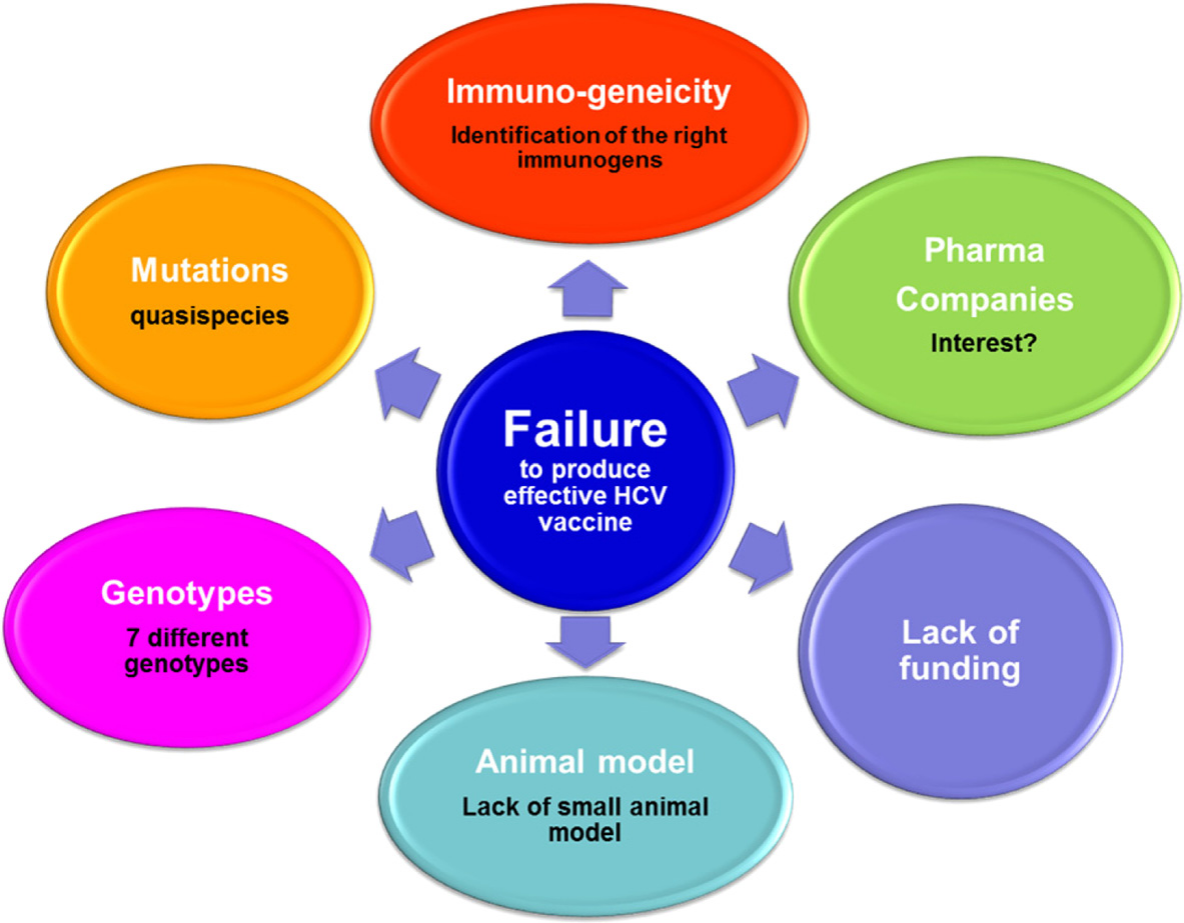
Causes of HCV vaccine failures

Our current knowledge of HCV spontaneous clearance is incomplete. However, natural resistance to infection exists and continues to provide optimism to researchers attempting to develop effective HCV vaccines [17, 18]. The high viral production rate and the existence of seven different HCV genotypes and quasi-species has hampered the design of an effective universal vaccine. Several other factors also contribute to the lack of an effective HCV vaccine until now. Figure 3 summarizes the known factors that contribute to the difficulties of producing an effective HCV vaccine including the shortage of funds for vaccine studies, the modest interest of pharmaceutical companies and lack of a small and convenient animal model. All these factors contribute to the failure in the development of an effective universal vaccine against HCV. On the other hand, there are great advances in the development of newly direct acting antivirals (DAAs) for the treatment of HCV infection with high cure rates. However, due to their high cost, there is a limited access to these new drugs in many parts of the world, reviewed elsewhere [19]. To this end, there is a great need for an effective pan- genotypic HCV vaccine.

Although HCV neutralizing antibodies have been identified, these antibodies are isolate-specific and do not correlate well with viral clearance [20]. In the absence of antibodies to HCV (anti-HCV) or detectable viremia, HCV-specific CMI may represent the sole host biomarker of exposure to this virus and may offer a protective mechanism against chronic HCV infection [10]. In this regard, what constitutes a protective immune response or correlate of protection against HCV infection is still vague. HCV-specific CMI was shown in exposed uninfected subjects [21–29] without viremia or seroconversion. This was documented among subjects such as family members living with two or more HCV infected subjects [24], sexual partners of acute HCV subjects [27], intravenous drug users (IVDU) [25, 26] and healthcare workers (HCW) [28, 29]. Interestingly, HCV transmission from seronegative blood donors was suggested to occur via cellular blood products [30]. The above data suggest that host immune responses likely determine the course of HCV infection. It may be possible to define the protective nature of HCV-specific CMI responses among these individuals, and whether these responses can be replicated by a vaccine.

## Review

### Innate immune responses to HCV infection

Similar to many viral infections, natural and adaptive immune responses are essential in controlling HCV infection. Hepatic innate immune reactions are linked to natural killer (NK) cells, NKT cells, dendritic cells, Kupffer cells and a quick IFN response mediated by infected hepatocytes. NK and NKT cells lyse infected cells by releasing granzymes and perforin. Additionally, these cells produce huge amounts of type-II IFN (represented by IFN-γ) and tumor necrosis factor alpha (TNF-α). HCV replication cycle leads to the assembly of double stranded RNA (dsRNA) intermediates that can trigger the expression of type-I IFN genes. In this regard, the host cells identify dsRNA through the pattern recognition receptors know as Toll-like receptor 3 (TLR-3) [31]. Recognition of dsRNA by TLR-3 leads to the activation of interferon regulatory factor 3 (IRF-3). IRF-3 induces the expression of IFN-β and interferon-stimulated genes (ISGs). IFNs act against HCV replication in host cells and protect the uninfected adjacent cells from infection with HCV by inducing the expression ISGs. In this regard, the role of TLR-3 in viral infections has been shown in knock-out mice that were unable to mount a response to measles virus infection [32]. Also, TLR-3 has been shown to mediate innate immune responses against HCV infection [33].

Early defense against viral infection requires the stimulation of type-I IFN, IFN-α and IFN-β. Mice that lack IFN-α and IFN-β fail in resolving HCV infection [34]. Additionally, genetic defects in Signal Transducers and Activators of Transcription-1 (STAT-1), which is involved in the IFN signaling cascade, also result in death of humans from viral disease(s) at an early age [35]. Among chimpanzees with acute HCV infection, type-I IFN induced the expression of dsRNA-dependent protein kinase R (PKR), 2’-5’ oligoadenylate synthetase (OAS) and Mx genes. These factors have an essential part in inhibiting HCV replication and inducing apoptosis of infected hepatocytes [36]. NK cells were, also, shown to have an essential part in eliminating HCV without measurable T cell responses in chimpanzees [37].

### Cellular immune response to HCV infection

Following acute infection with HCV, 15–50 % of infected people will spontaneously clear their infection [38]. Several studies suggest that successful viral clearance depends on the coordination of multiple arms of the immune system. Natural and specific immune responses play an essential part in this process, which has been reviewed elsewhere [39]. Vigorous and broad adaptive immune responses have been identified in acutely infected individuals and these responses endure among those who clear the virus. By contrast, persistent infection correlates with weak, often unnoticeable T-cell responses [40].

The adaptive immune response includes two major types of effector mechanisms: cellular responses comprising CD4 T helper (Th) cells and cytotoxic CD8 T lymphocytes (CTL); and humoral responses consisting of antibodies produced by HCV-specific B cells. Recognition of a specific viral epitope/protein is required for both adaptive immune effector mechanisms, which can target any HCV protein. However, only some B cell epitopes localized on the viral envelope or capsid proteins can induce HCV neutralizing antibodies and efficiently prevent the binding and entry of the virus, i.e., prevent HCV infection. Activated dendritic cells can present HCV antigens to specific Th cells that respond by proliferation and production of cytokines such as interleukin (IL)-2, IFN-γ or IL-4. Th cell activation and cytokine production is required for the development of CTL. Ideally, stimulated CTL found in the liver will lyse HCV-infected liver cells by cytolytic and non-cytolytic mechanisms [41].

Th and CTL are essential for the control of HCV infection in vivo. In this regard, the presence of strong T cell responses to the virus is a common characteristic among patients who clear their acute HCV infection. However, strong CD4 and CD8 responses may also occur in those who go on to chronic HCV infection. T cell responses are usually weak among HCV chronic carriers. The part played by the HCV-specific T cell response in controlling HCV infection is reinforced by several observations. First, the appearance of HCV-specific T cells in primary infection coincides with the decline in HCV viral load. Additionally, intrahepatic virus-specific CTL responses correlate with HCV clearance and with hepatic inflammatory response [42]. Second, a strong association between certain Class I and Class II alleles of human leukocytic antigen (HLA) and clearance of HCV infection has been reported. In this regard, HLA Class-I A3 and B27 alleles were shown to associate with protection and HCV clearance mediated by a response to dominant CD8 T cell epitopes [43]. Third, studies in chimpanzees demonstrated that T cell responses were essential for the HCV clearance/persistence process [12, 13].

Although HCV-specific T-cells can be more numerous in the peripheral blood mononuclear cells (PBMCs) of chronic patients than in recovered subjects, the T-cells in chronic subjects display a reduced proliferative response. T-cell lines derived from chronic HCV patients also displayed lower HCV-specific cytotoxicity when compared with cell lines derived from recovered subjects. Ex vivo IFN-γ production and the proliferation of HCV-specific cells were also defective among chronic patients. This defect could not be reversed by in vitro stimulation with IL-2 and HCV peptides. Additionally, the impairment of cytokine synthesis, cytotoxic function and HCV-specific T cell proliferation among viremic patients was associated with weak in vitro Th responses [44]. The highest frequency of HCV-specific response measured by IFN-γ Enzyme-linked immunospot (ELISPOT) assay were found within the first 8 weeks following diagnosis of HCV infection [44]. Moreover, subjects who spontaneously cleared their acute infection had a greater and broader HCV-specific T cell response when compared to their counterparts who developed chronic infection. Importantly, subjects with chronic HCV infection failed to sustain these responses and their CMI responses dropped to undetectable levels only 1 year after diagnosis of acute infection. By contrast, subjects who spontaneously resolved their infection had detectable HCV-specific T cell responses, although reduced in magnitude, up to 12 months after diagnosis of acute infection. Antigen-specific IFN-γ production by CD8 T cells declined more quickly during acute HCV-infection among subjects who went on to chronic course of the disease compared with their spontaneous resolution counterparts. Also, the extent and breadth of CD8 T cell responses acted similarly [45].

There is a need for comparative studies of the HCV-specific memory T cells responses induced by natural infection and by treatment. The part played by the immune system in controlling the outcome of HCV therapy is debatable with reports showing an enhanced [46, 47], unaffected or declining [48, 49] immune response among individuals responding to IFN-based therapy. In this regard, treatment of HCV infection at early stages could rescue long-lived HCV-specific memory T cells [50–52]. Importantly, IFN-α therapy has been shown to rescue polyfunctional HCV-specific CTL; which persisted for up to one year after therapy discontinuation [52]. On the other hand, there are limited data examining the dynamics of HCV-specific CMI responses after treatment with the new DAAs. In this regard, HCV-specific CTL were of narrow specificity among chronic HCV-infected chimpanzees and were stable over time after successful treatment with two DAAs [53].

#### Role of CD4 T cells

CD4 T cells deliver essential “help” for innate, cellular and humoral immunity. T helper cells activate antigen presenting cells, provide costimulatory signals for B cells, and prime and sustain CTL responses. HCV-specific Th cells correlate with transient and long-lived viral control [54–56]. Viral clearance and liver inflammation is usually synchronous with the buildup of HCV-specific Th and CTL within 8–14 weeks following infection. Several reports have revealed the presence of strong Th responses in spontaneously resolving HCV infections [42, 57, 58]. In contrast to the poor and narrow responses shown in those with chronic HCV, a strong, broad, and Th1-biased Th response was found in subjects with self-limited infection [59]. PBMCs isolated from subjects with self-limited HCV infection showed a Th1 cytokine profile, while those from chronic subjects displayed a Th2 profile. This suggests that Th1 and not Th2 responses are associated with a successful control of the virus in the early phase of infection [60–63]. Additionally, serum levels of Th2 cytokines are increased in chronic HCV infection and decrease during IFN-α therapy [64]. The extent and specificity of Th responses also seem to be essential for the control of HCV infection. In this regard, vigorous and multi-specific T cell responses and a sustained proliferation ability in response to HCV antigens are dependable measures of a protective CMI during acute infection [44]. T cells from subjects with spontaneously resolving HCV infection recognized a mean of 10 out of 37 identified HCV epitopes. On the other hand, those from patients with HCV persistence recognized only one epitope at max [65]. Circulating HCV-specific CD4 T cells from spontaneous clearance subjects simultaneously targeted 4-14 epitopes in the structural and non-structural HCV proteins for up to several years after RNA disappearance from the serum [65]. In summary, the above reports show that strong, broad, and Th1- biased responses were identified in spontaneous clearance subjects while poor and narrow responses were found among those who developed chronic infection [59, 66].

#### Role of CD8 T cells

The effector functions of CTL include two overlapping mechanisms: the killing of target cells and the non-cytolytic production of antiviral cytokines. An IFN-γ-mediated non-cytolytic pathway facilitates viral elimination, with >95 % of HCV replication inhibition happening at a low effector to target ratio [67]. Reports from animal studies, also, showed that CTL migrate to the liver and contribute to controlling HCV infection. The buildup of virus-specific CTL in the liver synchronizes with increased liver enzyme levels and with a transient decline in serum HCV-RNA levels [54, 68]. Like Th responses, the major player of spontaneously resolved HCV infection is a broad and multi-specific CTL response [42, 69–71]. Contrasting this observation, CTL responses are weak or target fewer epitopes in subjects having chronic infections [42, 66, 72].

#### Role of T regulatory cells in HCV infection

Regulatory T (T_reg_) cells are a subpopulation of T-cells that play an essential role in sustaining immune homeostasis and the balance between tissue damage and immune protection. T_reg_ cells were proposed as a possible mechanism for controlling HCV-specific responses [73–75]. Chronic HCV cases have an increased frequency of T_reg_ cells compared to controls and the T_reg_ cells negatively correlated with the degree of inflammation [76–78]. The higher frequency of T_reg_ cells may also explain the weak HCV-specific T-cell responses in chronic HCV patients [79]. There is also some evidence that chronic HCV patients may harbour more T_reg_ cells in their peripheral circulation [80] and in the liver than those who are uninfected [81]. Thus, T_reg_ cells appear to assist in the maintenance of chronic infection by inhibiting anti-HCV responses and, therefore, attenuating the intrahepatic tissue-damaging response to infection [79, 82].

### CMI responses without viremia or seroconversion in high risk subjects

1. Health care workers (HCW)

HCW are at an increased risk of HCV infection during their work [83, 84]. The risk of getting HCV infection from a contaminated needle is estimated at 0–5 %, or approximately 10-times higher than the risk of HIV infection via a comparable event [85, 86]. In general, the occupational risk of acquiring HCV infection by a surgeon is below 0.03 %/year. This is true even when the surgeon serves subjects with a high prevalence of HCV infection [87]. The probability of HCV infection is likely dependent on several features including viral load, time and mode of injury. However, there is no scoring system for the assessment of the risk of HCV infection after occupational exposure [28].

The detection of both anti-HCV antibodies and/or HCV-RNA are usually used for the clinical determination of HCV infection. Anti-HCV seropositivity in the absence of HCV-RNA indicates past HCV infection. Importantly, HCV-specific T cell responses were documented among individuals with self-limited HCV infection [42, 58, 65]. Additionally, HCV-specific T cell responses were reported among seronegative, aviremic subjects [21, 24, 25, 88, 89]. These studies detected HCV-specific T cells that reacted with different HCV proteins by the secretion of cytokines such as IFN-γ.

Transient viremia without HCV-antibody seroconversion was proposed as an explanation for the low incidence of new HCV infections among HCW [90] and the clearance of infection among other populations supposedly exposed to low levels of HCV [23, 24, 26–28, 88, 91–93]. More than 50 % of 52 seronegative, aviremic HCW had strong HCV multi-specific CMI responses suggesting clearance of low level HCV infections. These HCW were at a high risk of HCV infection by providing healthcare to a high HCV prevalence patient cohort [29]. These responses could arise from transient infection(s) with low titers of HCV-RNA that did not induce the production of anti-HCV, as reported among humans [22, 93, 94] and chimpanzees [37, 95]. The above data suggest that relying only on the detection of HCV antibodies to identify past exposure to the virus can lead to a substantial underestimation of prior exposure to the virus especially in endemic countries or among high-risk populations. Unfortunately, the protective nature of these responses and whether they protect these HCW are difficult to prove in humans. This difficulty can be ascribed to safety and ethical issues regarding the challenge of humans with live viruses.

Interestingly, a recent trial in four monkeys suggested that exposures to sub-infectious doses of HCV actually suppresses T cell responses upon subsequent acute infection [96]. On the other hand, brief low viremia had been documented in one HCW. This HCW remained seronegative and aviremic for more than a year of follow-up after transient viremia [90]. A similar observation was also documented among other HCW in Egypt [97, 98]. These data suggest the exposure to and clearance of HCV infection without antibody seroconversion. These seronegative, aviremic HCW are frequently exposed to HCV through occupational exposure. The CMI responses found among these HCW may protect them from HCV infection. An analogous phenomenon was documented in IVDU who cleared primary HCV infections and were protected against consequent exposure to HCV. This protection was largely due to T cell responses [10]. The above data demonstrate HCV-specific T-cell responses in exposed seronegative, aviremic individuals [21–27, 99] including HCW in Europe [28, 89] and Egypt [29, 100] and seem to be in a clear contrast to the trial performed in monkeys [96]. If these CMI responses protect these individuals, they will certainly impact the plans for HCV vaccine development, the determination of past-exposure to the virus and disease burden in different communities.

Evidence of HCV-specific T-cells producing IFN-γ was shown among HCWs caring for chronic HCV patients without any documented needle stick incidents [89]. HCV-specific T-cell secretion of IFN-γ was determined for pooled HCV peptides from the core region in 10 healthy aviremic HCWs with ≥7 years of healthcare experience, and 30 HCV chronic subjects. Patients with chronic HCV infection had a lower frequency of IFN-γ spot forming cells (SFCs) than the HCW. Physicians and nurses could have some exposure to HCV antigens that induce IFN-γ production by T-cells regardless of the prophylactic precautions undertaken while caring for chronic HCV patients. This could be related to continuous exposure of the immune system to HCV antigens. The exact route of exposure to/transmission of HCV infection in these individuals that leads to activation of T cells is mysterious and remains a subject of dispute [21, 27]. These routes may include constant exposure to the virus as previously suggested [21], perhaps via skin micro-lesions or an unprotected mucosa [101, 102], which happens during daily patient care. Although these HCW had regular laboratory monitoring, unreported or under estimated needle stick injuries cannot be excluded as a source of occupational exposure to HCV [103]. Other routes of transmission are likely present among immunocompromised and immunocompetent subjects. This was shown in hospitalized cancer patients without a known infection risk who were diagnosed positive for HCV antibodies and RNA [101, 104, 105].

2. Household contacts

Several studies reported HCV-specific CMI without viremia or seroconversion among household contacts. In one study, CMI was examined in persons exposed to HCV without evidence of HCV infection. These persons were living in a rural community in Egypt; where anti-HCV prevalence was 24 %. Thirteen of 71 (18 %) seronegative subjects with a high-risk exposure to HCV (living with ≥2 HCV-infected patients) and only one of 35 (2.9 %) seronegative low-risk subjects (no HCV-infected subjects living in the household) had detectable CMI [24]. The authors speculated that the subjects who were seronegative and showed CMI-positive responses had a transient very mild infection, probably due to exposure to low-dose(s) of the virus which was subsequently cleared. The presence of a prior HCV infection is supported by the fact that the majority of the detected responses were to non-structural HCV proteins; which is an indication of HCV replication within these subjects. Additionally, a substantial proportion of HCV-seronegative aviremic Egyptian children at risk of infection developed broad HCV-specific CMI. These responses were suggested as a possible protective mechanism in these children against the development of chronic infection [88]. Moreover, strong HCV-specific CMI responses were demonstrated among seronegative, aviremic children born to mothers infected with HCV 3–8 years after birth [99]. The children with transient viremia after birth had the strongest IFN-γ responses to HCV antigens, particularly the NS3/NS4 antigens, with up to 80 % responding to more than one HCV antigen. In another study, CTL responses against both the structural and non-structural HCV epitopes were detected in 24.1 % of healthy family members who were constantly exposed to chronic HCV persons (18). A similar phenomenon was reported among sex-workers repeatedly exposed to human immunodeficiency virus (HIV), without detectable viremia or antibodies. These sex workers developed strong HIV-specific CMI responses against HIV-1 antigens [106]. HIV-specific CMI was suggested as a protective mechanism in these subjects against HIV-infection, potentially leading to a quick control of the virus prior to the development of antibody responses. Similarly, some household contacts of HCV-infected patients or IVDU with high risk exposures to HCV were shown not to develop apparent infection despite repeated exposure to HCV [107].

3. Sexual contacts and spouses

HCV-specific CMI responses in seronegative sexual partners of chronic HCV patients was examined [92]. A positive HCV-specific CMI response was documented in the PBMCs of four subjects with occult HCV infection without the detection of HCV antibodies. The authors suggested that these CMI responses are biomarkers for prior exposure and recovery from HCV, and ongoing occult infection [92]. Sexual contacts of acute HCV patients were tested with a set of 18 Class-I-restricted peptide antigens and recombinant genotype 1 proteins [27]. A substantial number of exposed subjects who remained persistently aviremic and antibody negative developed both Th- and CTL-restricted responses which were similar in breadth and strength to that of subjects with primary and self-limited HCV infections.

HCV-specific CMI against the core and NS3 proteins of HCV genotype 1 were characterized in 32 seronegative, aviremic individuals without risk of exposure to HCV, 33 exposed seronegative, aviremic individuals and 20 uninfected individuals living with chronic HCV patients [108]. Twenty percent of apparently uninfected subjects had measurable HCV-specific CMI. This frequency is higher than prior estimates of HCV prevalence in developed countries. These data are consistent with spontaneously cleared primary HCV infections or infection that remained undetectable by traditional testing methods.

4. IVDU and prisoners

High-risk prisoners showed HCV-specific immune response without seroconversion and may have a higher probability of HCV clearance [22]. The majority of 40 high risk seronegative, aviremic long-term IVDU had HCV-specific CMI responses [109]. Fifty eight percent of exposed uninfected IVDU produced IFN-γ in response to HCV antigens compared with only 19 % of 21 control subjects. These responses were broad and targeted several HCV proteins, eliminating cross-reactivity to other antigens as a reason for these responses. These responses may denote an imprint for HCV exposure without viremia or antibody seroconversion. The same group later demonstrated weak IFN-γ responses to both structural and non-structural HCV antigens that were significantly higher among exposed uninfected IVDU compared to healthy controls. Among exposed seronegative, aviremic cases, individuals who were not in rehabilitation demonstrated a significantly higher frequency of IFN-γ producing cells in response to HCV antigens when compared with those who stopped injections [110]. Ongoing injecting behavior in the community or in prison was suggested as a primer for these T-cell responses. Moreover, upon longitudinal follow-up, exposed uninfected subjects continuing to inject drugs were more likely to maintain a detectable IFN-γ response when compared with those who stopped injections. It was suggested that continued drug injection is crucial for maintaining HCV-specific CMI, and that these responses are lost within months of stopping injections [110]. Similar reports documented broad HCV-specific T-cell responses among high-risk, seronegative aviremic IVDU [23, 25, 26].

5. Hemodialysis patients

The HCV-specific T-cell functional profile was examined among high-risk seronegative, aviremic hemodialysis patients [111]. Of seventy seven hemodialysis seronegative, aviremic patients, 11.3 % displayed HCV-specific CMI responses. Occult HCV infection was not a cause of this CMI response as proven by nested RT-PCR. Interestingly, based on the cytokine profile, two unique memory T cell populations were found in these patients. These included a polyfunctional population and another population with a dominant TNFα production [111].

#### Theories behind the existence of CMI responses without viremia or seroconversion

There are multiple possibilities for the development of T cell responses in seronegative, aviremic subjects. These include occult HCV infection with little viral replication [112, 113], cross-reactivity to heterologous epitopes [114, 115], brief viral replication that does not lead to antibody seroconversion [22, 95] and the loss of anti-HCV antibodies following clearance of the virus [15]. However, it is not clear why only HCV-specific T cells, but not B cells, are primed in seronegative, aviremic individuals.

The existence of antigen-specific cellular responses in some seronegative subjects may be explained by the presence of cross-reactive T-cells responding to antigens from unrelated organisms. In this regard, cross-reactive Th cells against epitopes of diverse herpes viruses was reported among humans [116]. Additionally, another study reported the presence of cross-reactive CTL responses between influenza A and HCV [115]. The existence of a few HCV-peptide specific T cells among humans does not necessarily indicate previous exposure to HCV. Also, cross-reactivity with other infectious agents can certainly affect the overall profile of HCV-specific T cells [117]. In this regard, the viremia level and HCV-specific immune responses were examined following viral inoculation of chimpanzees [95]. Infective HCV doses of 1–10 RNA (+) virions induced detectable CMI responses without viremia or seroconversion [95]. Another study also suggested that pre-existing CMI responses led to a more rapid expansion of adaptive immune responses upon subsequent exposure to infectious doses of HCV and a reduced frequency of chronic hepatitis [118]. Importantly, cross reactivity alone can not explain the HCV-multi-specific responses reported among many HCW [29].

## Summary

HCV spontaneous clearance remains poorly understood. However, natural protective immunity against the virus was documented in humans and chimpanzees. Host-specific CMI responses seem to have an essential role in this process. The high viral replication rate and the existence of seven HCV genotypes and quasispecies hamper the development of a universally effective vaccine. Several other factors also contribute to the unavailability of an effective HCV vaccine. Although neutralizing antibodies have been identified, they are isolate-specific and poorly correlate with viral clearance. By contrast, antigen-specific Th cells correlated with transient HCV viremia and long-lasting viral control. Vigorous, broad, and Th1-biased responses were identified among those individuals that resolve HCV infection, contrasting with the weak and narrow responses found among chronic subjects.

In the absence of anti-HCV antibodies or detectable viremia, CMI may represent the sole marker of host contact with HCV and may offer a protective mechanism against chronic hepatitis caused by this virus. HCV-specific CMI was documented among exposed subjects without viremia or seroconversion in several populations e.g., HCW, family contacts, prisoners, sexual partners, and IVDU. These data suggest the exposure to and clearance of HCV infection without seroconversion. Although direct challenge experiments cannot be performed in humans, it is possible that long-lasting seronegative persistence in fertile discordant couples will be able to prove, in the near future, the multiple exposure and the immune-protection of the seronegative partners as well as the part played by the immune response in protection from HCV infection. Further support will be provided by the seronegative, aviremic HCW constantly exposed to HCV antigens. The CMI responses mounted in these subjects can play a crucial role in their protection from HCV infection. An equivalent phenomenon is present among IVDUs who spontaneously resolve a primary HCV infection and are resistant to secondary HCV exposures. This protection correlates well with CMI responses.

Identification of host protective immune responses in subjects who spontaneously clear their HCV infection, and the longevity, magnitude, and breadth of the response; particularly in seronegative, aviremic subjects; is essential for the detection of prior exposure to HCV, understanding its natural history, and undertaking measures for its prevention. If these responses are protective, then similar CMI responses need to be simulated by future HCV vaccines. Unfortunately, the protective nature of these responses and whether those HCV-specific T cells in these seronegative, aviremic groups were able to induce viral clearance are difficult to prove in humans. Correlates of protection could be closely monitored among these seronegative, aviremic cohorts.

## Conclusions

To date, there is no licensed HCV vaccine. The mechanisms behind successful HCV clearance suggest the coordination of multiple arms of the immune system, with CMI playing an essential role in this process. HCV-specific CMI has been reported among several high risk subjects without viremia or seroconversion suggesting clearance of prior infection(s) with HCV. Further studies are needed to examine the longevity of HCV-specific CMI responses in seronegative, aviremic subjects; particularly HCW; and to determine the dominant epitopes in the responding antigens. It may be possible to define the protective nature of HCV-specific CMI responses in humans, and whether these responses can be replicated by a vaccine.

## Abbreviations

Anti-HCV: antibodies to hepatitis C virus
CMI: cell-mediated immunity
CTL: cytotoxic T lymphocyte
DAAs: direct acting antivirals
dsRNA: double stranded RNA
ELISpot: enzyme-linked immunospot assay
HCC: hepatocellular carcinoma
HCV: hepatitis C Virus
HCW: healthcare workers
HIV: human immunodeficiency virus
IFN: interferon
IL: interleukin
IRF-3: interferon regulatory factor 3
ISGs: interferon-stimulated genes
IVDU: intravenous drug users
NK: natural killer
OAS: 2’-5’ oligoadenylate synthetase
PKR: protein kinase R
SFCs: spot forming cells
SNP: single nucleotide polymorphism
STAT: signal transducers and activators of transcription
Th: T helper
TLR-3: toll like receptor 3
TNFα: tumor necrosis factor α
T_reg_: regulatory T cells
